# METTL3-mediated pre-miR-665/DLX3 m^6^A methylation facilitates the committed differentiation of stem cells from apical papilla

**DOI:** 10.1038/s12276-024-01245-8

**Published:** 2024-06-03

**Authors:** Tingjie Gu, Rong Guo, Yuxin Fang, Ya Xiao, Luyao Chen, Na Li, Xingyun Kelesy Ge, Yijia Shi, Jintao Wu, Ming Yan, Jinhua Yu, Zehan Li

**Affiliations:** 1 Jiangsu Key Laboratory of Oral Diseases, Nanjing Medical University and Department of Stomatology, Nanjing Medical University, Nanjing, China; 2 Department of Endodontics, Affiliated Hospital of Stomatology, Nanjing Medical University, Nanjing, China; 3 Faculty of Dentistry, The University of Hong Kong, 34 Hospital Road, Hong Kong SAR, China

**Keywords:** Stem-cell differentiation, Mesenchymal stem cells

## Abstract

Methyltransferase-like 3 (METTL3) is a crucial element of N6-methyladenosine (m^6^A) modifications and has been extensively studied for its involvement in diverse biological and pathological processes. In this study, we explored how METTL3 affects the differentiation of stem cells from the apical papilla (SCAPs) into odonto/osteoblastic lineages through gain- and loss-of-function experiments. The m^6^A modification levels were assessed using m^6^A dot blot and activity quantification experiments. In addition, we employed Me-RIP microarray experiments to identify specific targets modified by METTL3. Furthermore, we elucidated the molecular mechanism underlying METTL3 function through dual-luciferase reporter gene experiments and rescue experiments. Our findings indicated that METTL3^+/−^ mice exhibited significant root dysplasia and increased bone loss. The m^6^A level and odonto/osteoblastic differentiation capacity were affected by the overexpression or inhibition of METTL3. This effect was attributed to the acceleration of pre-miR-665 degradation by METTL3-mediated m^6^A methylation in cooperation with the “reader” protein YTHDF2. Additionally, the targeting of distal-less homeobox 3 (DLX3) by miR-665 and the potential direct regulation of DLX3 expression by METTL3, mediated by the “reader” protein YTHDF1, were demonstrated. Overall, the METTL3/pre-miR-665/DLX3 pathway might provide a new target for SCAP-based tooth root/maxillofacial bone tissue regeneration.

## METTL3 dysregulation leads to root dysplasia and bone loss

Dentition defects, or missing teeth, can cause long-term health issues and are typically treated with implant denture restoration, a method that often has complications. Recent studies suggest using dental stem cells for treatment. A study by Nanjing Medical University investigated the role of a molecule, METTL3, in the development of stem cells from the apical papilla (SCAPs), a type of dental stem cell. They discovered that METTL3 helps SCAPs develop into odonto/osteogenic cells, important for tooth and bone growth. This implies that METTL3 could enhance treatments for missing teeth. The study also found a regulatory network involving METTL3 and other molecules, which could be significant in future research.

This summary was initially drafted using artificial intelligence, then revised and fact-checked by the author.

## Introduction

Dentition defects are a prevalent clinical disease characterized by an uneven distribution of missing teeth in either the upper or lower jaw. Apart from its diverse effects on mastication, articulation, and aesthetics, this condition is also linked to the development of numerous chronic ailments in individuals^1^. Currently, implant denture restoration, the primary approach employed, is subject to stringent criteria and contraindications and commonly results in complications such as peri-implant inflammation and implant fracture. These deficiencies negatively affect the overall prognosis and efficacy of long-term oral implant repair^2,3^.

Recently, notable advancements have been made in the area of dental stem cell research, resulting in promising treatment approaches for dentition defects. Notably, the transplantation of stem cells from the apical papilla (SCAPs) along with hydroxyapatite/tricalcium phosphate scaffold materials successfully facilitated the generation of biological roots in the alveolar fossa of small pigs^4^. Additionally, in SD rats, implantation of a combination of deciduous tooth stem cells and dentin matrix materials into the mandibular defect area was performed. After 8 weeks, the regenerated tissues exhibited a closely arranged and orderly structure closely resembling the natural periodontal ligament fibers surrounding the implant^5^. Despite these findings indicating the potential for successful regeneration of biological tooth roots through dental stem cells, importantly, the success rate of this approach is lower than that of traditional implant methods. One of the primary factors contributing to this outcome is the absence of precise regulation of the directional differentiation of dental stem cells^6,7^.

From the perspective of tissue affinity, apical papilla tissue plays a pivotal role in the genesis of dental roots. During the early stage of dental root formation in miniature pigs, the surgical extraction of apical papilla tissues notably inhibited the growth of dental roots, despite the preservation of dental pulp tissues^8,9^. Studies have shown that SCAPs exhibit elevated proliferative activity, versatile differentiation potential, and robust self-renewal capability^10,11^. These biological characteristics are closely associated with the formation of tooth roots. In contrast to dental pulp stem cells (DPSCs), SCAPs demonstrate increased proliferative activity and show a greater potential for tissue regeneration. Consequently, SCAPs are pivotal progenitor cells for biological tooth root regeneration^8^. Our previous studies revealed that the biological processes involved in the committed differentiation of SCAPs are regulated by a complex and intricate network^12–14^.

N6-methyladenosine (m^6^A) is a common alteration present in eukaryotic mRNAs or noncoding RNAs^15^. In mammals, the regulation of mRNA by m^6^A is primarily facilitated by methyltransferase complexes (writers) comprising METTL3, METTL14, and WTAP, and this process is reversed by demethylase complexes (erasers), including ALKBH5 and FTO. Subsequently, “reader” proteins such as YTHDF1 and YTHDF2 identify these relevant m^6^A modifications, which contributes to diverse biological processes such as RNA stability, transcription translation, and alternative splicing^16–18^. METTL3 contains a functional methyltransferase region that is essential for facilitating the transformation of adenosine (A) into m^6^A. The notable involvement of METTL3 in the differentiation and self-renewal of stem cells has been extensively documented^19–21^. However, the precise impact of METTL3 and METTL3-mediated m^6^A modification on the committed differentiation of SCAPs is unclear.

This study highlights the importance of METTL3 in the differentiation of SCAPs into odonto/osteogenic cells. Furthermore, through bioinformatics analysis and model animal techniques, we investigated the molecular mechanisms by which METTL3 potentially affects the m^6^A modification of pre-miR-665/DLX3.

## Materials and methods

### Ethics approval

All animal procedures conducted in this study were in accordance with the regulations set forth by the ethics committee of the National Science and Technology Commission, and experiments involving human tooth samples also met the requirements of the Ethics Committee of Nanjing Medical University (2022-429).

### Cell culture

The apical papilla tissues were obtained from premolars donated by disease-free individuals at the Affiliated Stomatological Hospital of Nanjing Medical University.

The human apex tissue was meticulously separated from the underdeveloped root. The collected tissues were sliced into 1 mm^3^ fragments and then subjected to digestion in a solution comprising 3 mg/mL collagenase type I and 4 mg/mL dispase (Sigma, USA) at 37 °C. The resulting cells were then seeded in 60 mm culture dishes with α-MEM (Gibco, USA) containing 10% fetal bovine serum (Gibco, USA), 100 μg/mL streptomycin, and 100 U/mL penicillin (Gibco, USA) in a humidified 5% CO_2_ incubator at 37 °C. SCAPs at 3–5 passages were used for the experiments. SCAPs were cultured in osteogenic medium (OM) at 50–60% confluence. OM was composed of conventional growth medium, 10 nM dexamethasone, 100 μM ascorbic acid, and 2 mM 2-glycerophosphate (Sigma, USA).

### Trilineage differentiation of SCAPs

SCAPs were cultured in OM for 21 days. After fixation with 70% ethyl alcohol for 15 min, the SCAPs were stained with alizarin red (Oricell, China) for 30 min. Alizarin red was dissolved in 10% cetylpyridinium chloride (CPC) for quantification. The final calcium concentration was normalized to the total protein content.

SCAPs were cultured in adipogenic differentiation medium (Oricell, China). When the density of the cells reached 90–100%, OriCell adipogenic differentiation medium A solution and B solution were alternately added to the medium. After 21–30 days, Oil Red O staining was conducted to visualize lipid droplets.

SCAPs were cultured in a 15 mL sterile tube for 3D pellet culture to induce chondrogenic differentiation (Oricell, China). Approximately 25 days later, the pellet was fixed and embedded in OCT compound at a thickness of 5 μm, followed by dying with Alcian blue.

### Cell transfection and lentivirus infection

METTL3 small interfering RNAs (siRNAs), si-YTHDF1, si-YTHDF2, si-YTHDF3, si-IGF2BP1, si-IGF2BP2, si-IGF2BP3, si-DLX3 and miR-665 mimics, mimic control (NC), and inhibitor and inhibitor control (INC) were designed by RiboBio Corporation. The oligonucleotide sequences used in the study were presented in Supplementary Table 2. Once the cell density reached approximately 70%, the SCAPs were infected with a riboFECT^TM^ CP kit (RiboBio, China); GenePharma Company (China) provided recombinant lentiviruses that contained full-length METTL3 and a scramble control (NC). SCAPs were exposed to 1 mL of α-MEM supplemented with 10% FBS and 8 μg/mL polybrene (POL) for 10 h.

### Quantitative real-time PCR (qRT‒PCR)

RNA was extracted from SCAPs using TRIzol (Invitrogen, USA) and subjected to reverse transcription using a PrimeScript RT kit (Vazyme, China). qRT‒PCR was performed on an ABI 7300 real-time PCR system using the Universal ChamQTM SYBR Green quantitative PCR Master Mix (Vazyme, China). *GAPDH* and *U6* were utilized as internal controls for mRNA and miRNA, respectively. A Bulge-Loop miRNA qPCR Primer Kit (RiboBio, China) was used to measure the expression of pre-miR-665/miR-665. The levels of genes associated with odonto/osteoblasts were assessed using the 2^−ΔΔCt^ method, following a previously described protocol^12^. The primer sequences were presented in Supplementary Table 1.

### Western blotting

SCAPs were lysed in buffer for cell lysis (Beyotime, China) supplemented with PMSF proteinase inhibitor while being kept on ice. Then, the proteins were quantified using a Bradford protein assay kit (Beyotime, China). Protein samples (25 μg) were separated on a polyacrylamide gel, transferred onto a nitrocellulose membrane, and subsequently blocked with 5% fat-free dry milk at RT for 2 h. Afterward, the membrane was incubated with rabbit anti-RUNX2 antibody (Abcam, UK), rabbit anti-ALP antibody (Abcam, UK), rabbit anti-OSX antibody (Abcam, UK), rabbit anti-DMP1 antibody (R&D, USA), rabbit anti-DSPP antibody (Bioworlde, USA), rabbit anti-METTL3 antibody (Proteintech, China), rabbit anti-DLX3 antibody (Proteintech, China), or rabbit anti-GAPDH (Proteintech, Wuhan, China) antibody at 4 °C overnight. The membranes were incubated with secondary antibody for 1 h at RT. Finally, the results were quantified by determining the gray values with ImageJ software. We provided the raw data for the protein bands in the study (Supplementary Fig. 6).

### Detection of alkaline phosphatase (ALP) staining and activity

SCAPs were stained for NBT/NCIP using the staining kit protocol provided by Jiancheng, China. The cells were treated with 4% PFA for 30 min, followed by incubation in an alkaline solution at 37 °C for 30 min. ALP activity was measured using an ALP activity assay kit from Beyotime, a company based in China. The protein content of each sample was measured by utilizing a Bradford Protein Assay Kit. The normalized relative expression of each sample in comparison to the control was adjusted based on the total protein content.

### m^6^A dot blot and m^6^A quantification

The extracted RNAs were directly applied onto a Hybond-N^+^ membrane (GE HealthCare, USA) and subjected to UV crosslinking using a Stratalinker 2400 UV crosslinker. The membranes were then blocked with 5% nonfat dry milk at RT for 1 h and incubated overnight at 4 °C with an anti-m^6^A antibody (Cell Signaling Technology, USA). Dot blots were subsequently incubated with HRP-conjugated anti-rabbit immunoglobulin G (IgG) for 1 h before visualization using an imaging system. For determination of the total RNA content, the membranes were stained with 0.02% methylene blue (MB). The quantification of m^6^A RNA methylation was performed using the m^6^A RNA methylation quantification kit following the manufacturer’s protocols (Epigentek, USA). The percentage of m^6^A was measured using a microplate reader at an absorbance of 450 nm.

### MeRIP-qPCR

MeRIP-qPCR was employed to investigate the m^6^A modifications of target genes and pre-miRNAs/miRNAs following the manufacturer’s protocol (Epigentek, USA). Briefly, a suitable quantity of RNA was extracted, with approximately 10% reserved as the input control, while the remaining RNA was combined with the anti-m^6^A antibody and subjected to 90 min of rotation, followed by prewashing with affinity beads. After three washes, RNA purification solution and 100% ethanol were added to the samples following proteinase K digestion, and the samples were subsequently stored at −20 °C for subsequent experiments. The m^6^A enrichment in each sample was determined by normalization to the input.

### RNA stability assay

SCAPs were subjected to treatment with 5 µg/mL actinomycin D (ActD) (AbMole, USA) to inhibit RNA transcription. At 0, 2, 4, or 6 h following ActD treatment, RNA samples were extracted, and the level of *DLX3* was quantified via qRT‒PCR.

### Dual-luciferase reporter assay

HEK-293T cells were transfected with miR-665 or a negative control and subsequently transfected with luciferase plasmids (GeneChem, China). After a 48-h incubation period, luciferase activities were measured using a Dual-Luciferase Reporter Assay kit (Promega, USA).

### Generation and maintenance of METTL3+/– mice

The CRESPR/Cas9 system was used to establish a METTL3 knockdown mouse model (C57BL/6 N background), which was generated with the assistance of the experimental animal center of Nanjing Medical University. Briefly, exon 4 of METTL3 was the target of interest in the generation of METTL3^+/−^ mice. Donor vectors with exon 4 combined with Cas9 mRNA and gRNAs were coinjected into fertilized C57BL6/N eggs. Subsequently, the injected zygotes were transferred into the oviducts of pseudopregnant females to obtain F0 mice. The genotype of the transgenic mice was determined by extracting the tail genomic DNA for PCR analysis. The sequences of the METTL3^+/−^ mouse primers used were as follows: forward primer (F1): 5’-CAACAGTCAACGAAAGAACAGCAG-3’; reverse primer (R1): 5’-AGGGAATCAGAATCAAGATGGTAC-3’.

### In situ jawbone transplantation in Sprague‒Dawley rats

Female Sprague‒Dawley rats (8 weeks) were utilized for jawbone transplantation. The rats were subjected to anesthesia, and subsequent separation of nerves and muscles was performed to expose the edentulous region of the mandible. Surgical defects measuring approximately 2 × 2 mm were then created in the aforementioned edentulous area. Next, gelatin sponges containing SCAPs were transplanted into the defects, followed by meticulous suturing. After approximately 8 weeks, the rats were humanely sacrificed, and their mandibles were collected for further analysis. The Institutional Animal Care and Use Committee of Nanjing Medical University approved all the animal studies described herein (No. 2022429).

### Microarray analysis

Total RNA was extracted from the SCAPs in the METTL3-OE or NC-OE group on Day 7 of osteogenic differentiation following the manufacturer’s protocol (Arraystar, USA). The samples were incubated with m^6^A antibody for IP. The modified RNAs eluted from the immunoprecipitated magnetic beads were named “IP,” and the unmodified RNAs obtained from the supernatant were named “Sup,” followed by labeling with Cy5 and Cy3. An Arraystar Super RNA Labeling Kit was used to designate these RNAs as cRNAs on their own. The cRNAs were combined and hybridized onto the Arraystar Mouse Epitranscriptomic Microarray (8 × 60 K, Arraystar). An Agilent G2505C scanner was used to scan the arrays in two-color channels after washing the slides. Finally, the raw intensities of IP and Sup were normalized to the average of the log_2_- scaled spike-in RNA intensities.

### Immunofluorescence staining and immunohistochemistry assays

Transfected SCAPs were seeded in 96-well plates and cultured for 24 h. The cells were washed with PBS and fixed with 4% PFA for 30 min at RT. After PBS washes, the cells were incubated with 0.05% Triton-100 (Beyotime, China) for 20 min and then blocked with normal goat serum. Treatment with primary antibodies was conducted at 4 °C overnight, followed by secondary antibody labeling with fluorochrome (Invitrogen, USA) for 1 h in the dark. Immunofluorescence images were collected under an inverted fluorescence microscope (Leica, Germany).

The hard tissues were decalcified using a 10% EDTA solution for one month. Subsequently, the tissues were dehydrated, and paraffin sections were prepared. The slides were then subjected to antigen retrieval and treated with a primary antibody overnight after being exposed to gradient ethanol. The following day, a secondary antibody was applied and incubated for a period of 20 min, followed by Masson and H&E staining. Finally, gradient dehydration was performed, and the slides were mounted using neutral balsam. All images were captured using a fluorescence microscope manufactured by Leica, Germany.

### Statistical analysis

The Statistical Package for Social Sciences (SPSS) software 22.0 was used for the statistical analyses. GraphPad Prism software was used to analyze the data expressed as the mean ± SD from at least three independent experiments. Differences were compared mainly through one-way analysis of variance and Student’s test. A two-tailed *P* < 0.05 was considered to indicate statistical significance.

## Results

### METTL3 expression is positively correlated with odonto/osteoblast differentiation in SCAPs

**Fig. 1:**
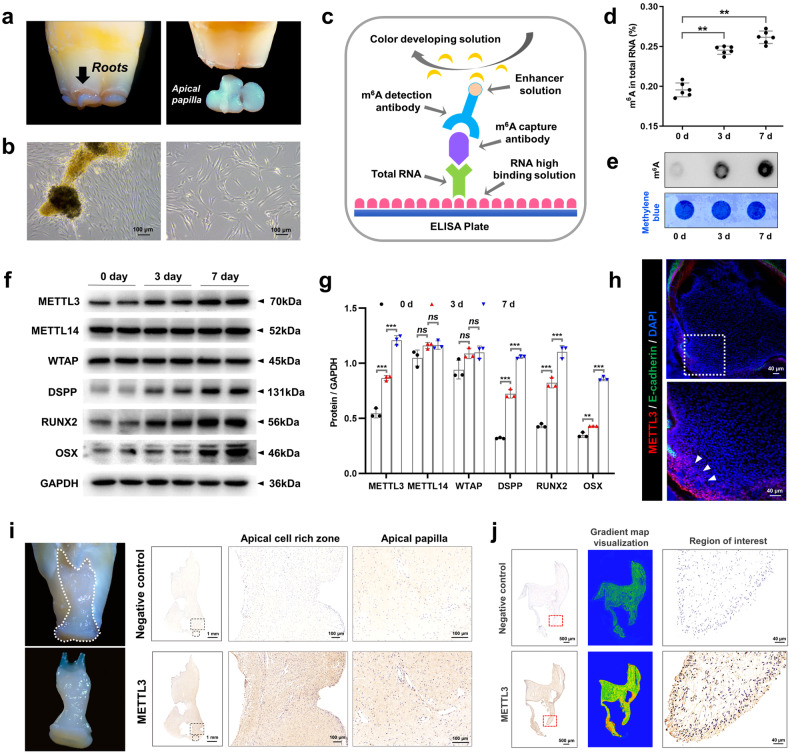
The expression of METTL3 is positively correlated with odonto/osteogenic differentiation. **a**, **b** SCAPs were isolated according to the literature. SCAPs displayed a typical spindle-like morphology in the original and third generations. Scale bar = 100 µm. **c**, **d** Annotation of the m^6^A RNA methylation quantification kit and the expression of m^6^A at 0, 3, and 7 days of mineralization; *n* = 6. **e** m^6^A dot blot showing an increase in global m^6^A levels during osteogenic induction in SCAPs at 3 and 7 days. *n* = 3. **f**, **g** Western blotting was performed to analyze the expression of METTL3, METTL14 and WTAP during SCAP odonto/osteogenic differentiation at 0, 3 and 7 days. *n* = 3. **h** Immunofluorescence staining was conducted on CD1 mice at postnatal Day 7. Scale bar = 40 µm. **i** Representative images of immunohistochemistry assays demonstrating the enrichment of METTL3 in the papilla tissue of human apical teeth. PBS was used as a negative control. Scale bar = 1 m; 100 µm. **j** Immunohistochemically stained samples of the METTL3-enriched zone in the papilla tissue of human apical teeth. PBS was used as a negative control. Scale bar = 500 µm; 40 µm. ns indicates no significance; ** indicates *P* < 0.01; *** indicates *P* < 0.001.

SCAPs were isolated and exhibited a characteristic spindle-like morphology (Fig. 1a, b). Flow cytometric analysis confirmed that the extracted SCAPs were undifferentiated stem cells derived from the mesenchyme, as these cells expressed CD29, CD73, CD90, and CD105 but did not express CD34 or CD45 (Supplementary Fig. 1). Additionally, the ability of SCAPs to differentiate into three lineages, namely, chondrogenic, osteogenic, and adipogenic lineages, was confirmed through Alcian blue staining, Alizarin red staining, and Oil red O staining, respectively (Supplementary Fig. 2). The results indicate that SCAPs have the ability to differentiate in multiple directions.

To assess METTL3-mediated m^6^A modification during odontogenic induction, we cultured SCAPs in odontogenic induction medium for 0, 3, or 7 days. The results demonstrated a gradual increase in the overall level of m^6^A during SCAP mineralization (Fig. 1c–e). Additionally, western blotting analysis revealed a progressive increase in METTL3 expression with prolonged induction time, accompanied by increased expression of odonto/osteoblast markers (DSPP/RUNX2/OSX). Conversely, the expression of the main “writer” proteins WTAP and METTL14 did not significantly differ (Fig. 1f, g). To observe the in vivo expression pattern of METTL3, we conducted immunofluorescence staining on postnatal Day 7 CD1 mouse samples. The findings demonstrated that METTL3 is primarily present in the apical papilla tissue of tooth roots (Fig. 1h). Additionally, immunohistochemistry assays confirmed that enriched localization was observed in the human dental papilla (Fig. 1i). METTL3 was also expressed in the apical tissue of third molars that displayed almost complete root development (Fig. 1j). In general, these findings indicate that METTL3-induced m^6^A alterations might affect the differentiation of odonto/osteoblasts in SCAPs.

### METTL3 knockdown (METTL3^+/−^) mice exhibit tooth root and bone dysplasia

**Fig. 2:**
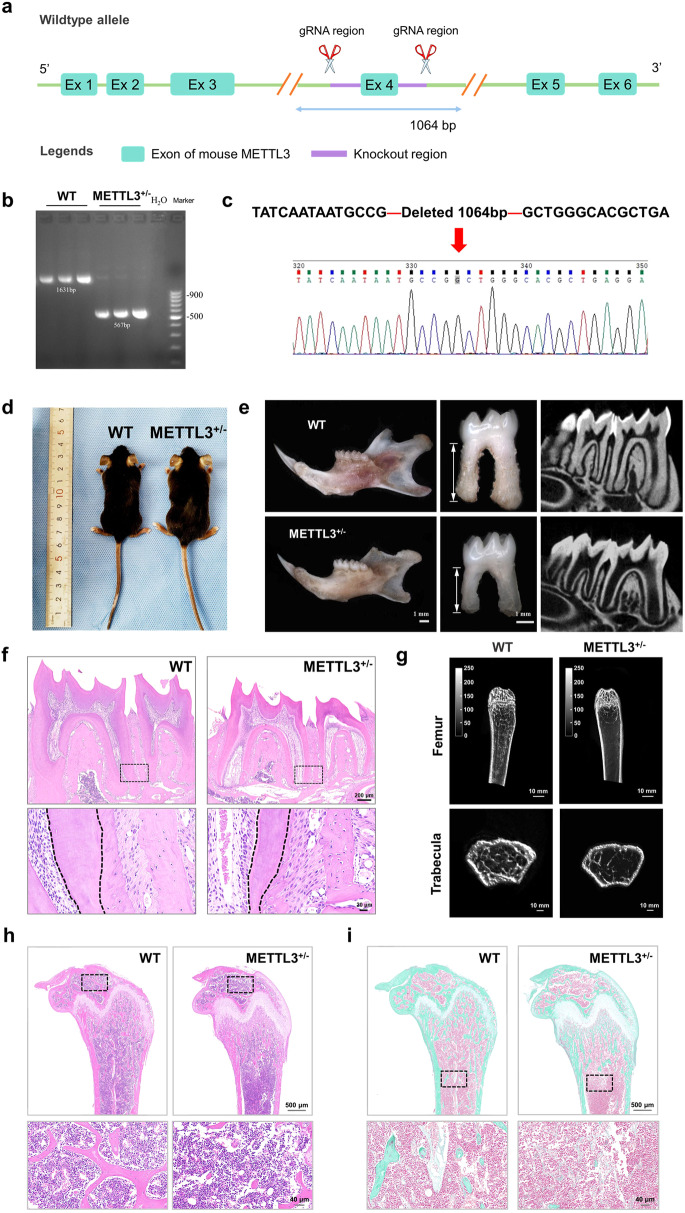
Negative impact of METTL3 knockout on bone mass and density and tooth root development. **a**–**d** Schematic illustration of the generation of METTL3^+/−^ knockout mice (C57BL/6 N) via a CRISPR/Cas9-mediated genome engineering strategy. Six exons were identified. Exon 4 was selected as the target site. Cas9 and guide RNA (gRNA) were coinjected into fertilized eggs for mouse production, followed by genotype identification of METTL3^+/−^ KO mice. **e** A reduction in root length and dysplasia of the tooth root in METTL3^+/−^ knockout mice compared to WT mice were observed via stereoscopic microscopy and micro-CT analyses. Scale bar = 1 mm. **f** Representative images of HE staining indicating that the width of the root dentin in the mandibular first molar was greater in METTL3 +/- mice than in WT mice. Scale bar = 200 µm; 20 µm. **g** Representative micro-CT images of the femoral metaphysis and entire proximal femur. Scale bar = 10 mm. **h**, **i** Representative images of HE staining and Golder staining showing that METTL3^+/−^ mice exhibited reduced bone formation relative to that of their WT counterparts. Scale bar = 500 µm; 40 µm.

To investigate the role of METTL3 in tooth root and bone development, we established a METTL3 knockdown mouse model using the CRISPR/Cas9 system. As METTL3 homozygous knockout (METTL3^-/-^) mice showed early embryonic lethality, we used METTL3 heterozygous knockdown (METTL3^+/−^) mice for subsequent experiments^22^. The genotype was initially confirmed through PCR and sequencing and further verified by western blotting (Fig. 2a–d, Supplementary Fig. 3a). H&E staining revealed no discernible differences in the size or morphology of the lung, spleen, kidney, liver, or heart between METTL3^+/−^ mice and wild-type (WT) mice (Supplementary Fig. 3b). Subsequently, stereoscopic microscopy and micro-CT analyses of mandibular first molars revealed a reduction in molar root length and root dentin width in METTL3^+/−^ mice compared to their WT littermates. Furthermore, the mandibular first molars of METTL3^+/−^ mice exhibited a significant reduction in dentin width, as evidenced by H&E staining (Fig. 2e, f). Additionally, the results obtained from micro-CT, H&E staining, and Goldner staining collectively indicated that METTL3 knockdown significantly decreased bone mass and bone formation (Fig. 2g-i). Taken together, the above results suggest the important and notable role of METTL3 in root development and bone formation.

### METTL3 positively regulates the odonto/osteogenic differentiation capacity of SCAPs

**Fig. 3:**
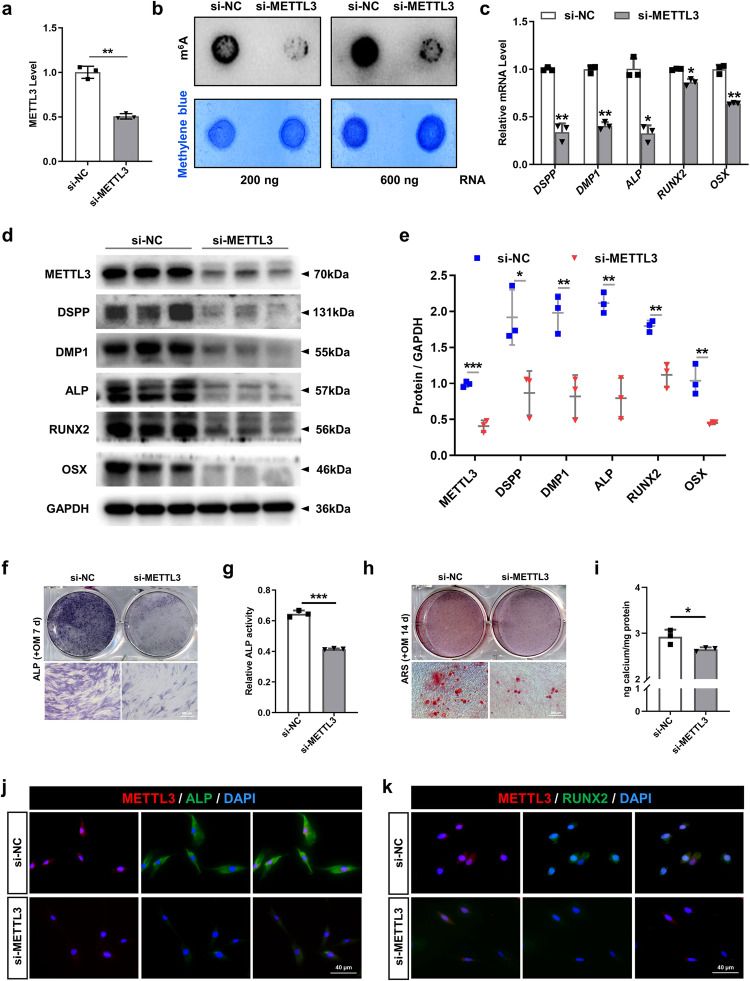
Silencing METTL3 inhibits the odonto/osteogenic differentiation of SCAPs. **a** The efficiency was measured by qRT‒PCR in SCAPs transfected with si-METTL3. **b** m^6^A dot blot assay showing the negative effects of METTL3 inhibition on the m6A content. **c** qRT‒PCR showed that the mRNA levels of *DSPP, DMP1, ALP, RUNX2* and *OSX* were decreased upon METTL3 silencing. **d**, **e** The protein expression of METTL3, DSPP, DMP1, ALP, RUNX2 and OSX decreased after METTL3 inhibition. **f**–**i** Representative images of ALP staining and ARS staining and relative quantification showing the negative effects of METTL3 silencing compared with si-NC treatment at 7 and 14 days in the mineralized medium of SCAPs. Scale bar = 200 µm. **j**, **k** Representative images of immunofluorescence staining for METTL3, ALP and RUNX2 in SCAPs after METTL3 silencing. Scale bar = 40 µm. *n* = 3, * indicates *P* < 0.05; ** indicates *P* < 0.01; *** indicates *P* < 0.001.

To assess the regulatory impact of METTL3 on the odontogenic/osteogenic differentiation of SCAPs, we suppressed METTL3 via transfection of specific small interfering RNAs (siRNAs) (Fig. 3a). As expected, m^6^A dot blot analysis revealed a decrease in the overall m^6^A level in SCAPs upon METTL3 knockdown (Fig. 3b). Furthermore, the expression levels of genes and proteins associated with odonto/osteogenic differentiation decreased, whereas METTL14 did not significantly change (Fig. 3c–e, Supplementary Fig. 3c). Reduced ALP staining and activity, as well as decreased ARS staining, revealed a diminished degree of odonto/osteogenesis and mineralization in SCAPs (Fig. 3f–i). Immunofluorescence staining analysis additionally demonstrated a significant reduction in the protein expression levels of RUNX2 and ALP in the SCAPs transfected with si-METTL3 compared to those in the si-NC group (Fig. 3j, k).

**Fig. 4:**
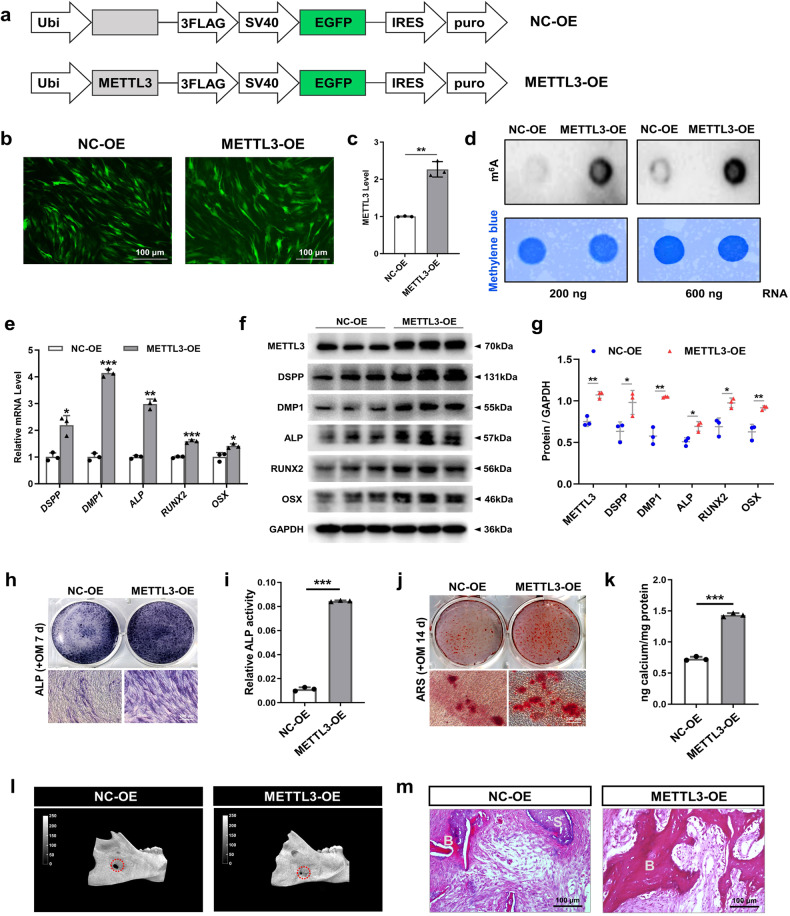
Overexpression of METTL3 promotes the odonto/osteogenic differentiation of SCAPs. **a** Structural diagram of lentivirus-mediated infection of SCAPs with METTL3-OE or NC-OE. **b, c** Immunofluorescence staining and qRT‒PCR were used to verify the efficiency of METTL3-OE and NC-OE infection at 72 h in SCAPs. Scale bar = 100 µm. **d** m^6^A dot blot assay showing that METTL3 overexpression increased m^6^A levels in SCAPs. **e** qRT‒PCR showed that METTL3 overexpression increased the mRNA expression of *DSPP, DMP1, ALP, RUNX2* and *OSX*. **f**, **g** Western blot analysis of METTL3, DSPP, DMP1, ALP, RUNX2 and OSX after METTL3 overexpression. **h**–**k** Representative images and relevant quantification of ARS-stained ALP staining and mineralized nodule formation. Scale bar = 200 µm. **l**, **m** Micro-CT and H&E staining were used to verify the role of METTL3 in regulating SCAP osteogenesis. S: scaffold; B: bone-like tissue; scale bar = 100 µm. *n* = 3, * indicates *P* < 0.05; ** indicates *P* < 0.01; *** indicates *P* < 0.001.

In contrast, the impact of METTL3 overexpression on the differentiation of SCAPs was investigated using recombinant lentiviruses to introduce METTL3 (METTL3-OE) into SCAPs (Fig. 4a–c). Overexpression of METTL3 led to elevated levels of m^6^A, as shown by the m^6^A dot blot assay (Fig. 4d). qRT‒PCR and western blotting revealed that the upregulation of METTL3 substantially increased the levels of odonto/osteogenic biomarkers (Fig. 4e‒g). ALP staining and ARS staining revealed a greater number of calcified nodules in the METTL3-OE group than in the NC-OE group, as further confirmed by increased ALP activity and CPC quantification analysis (Fig. 4h–k). To thoroughly examine the role of METTL3-mediated m^6^A modification in the process of bone regeneration, we generated a bilateral mandibular defect model in SD rats. As expected, compared with the control group, the METTL3-overexpressing SCAP group exhibited more bone-like structures, as shown by micro-CT and H&E staining (Fig. 4l, m). Collectively, these findings suggest that METTL3-mediated m^6^A modification potentially exerts a beneficial effect on the odonto/osteogenic differentiation of SCAPs.

### Pre-miR-665/miR-665 is a functional target of METTL3 in SCAPs

**Fig. 5:**
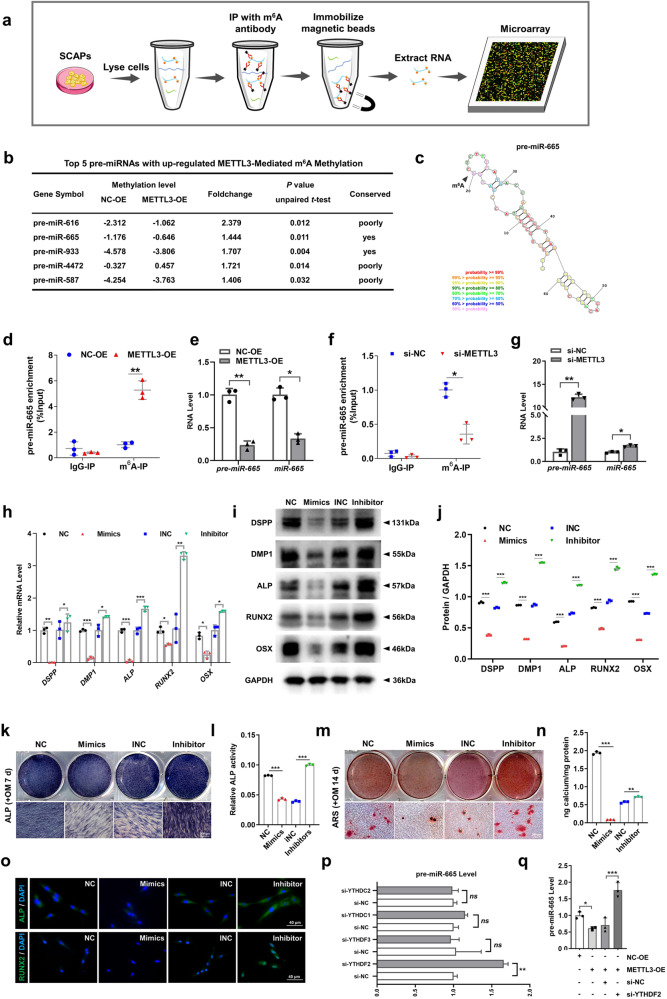
METTL3 alters the m^6^A methylation status of pre-miR-665 and the expression of pre-miR-665 and miR-665. **a** Flowchart of the m^6^A-RIP microarray analysis procedure used to identify the targets of METTL3. **b** The top 5 pre-miRNAs with upregulated METTL3-mediated m^6^A methylation are listed. **c** The combined sites of pre-miR-665 and m^6^A methylation are labeled. **d** MeRIP-qPCR showed the positive effect of METTL3-OE on pre-miR-665 methylation by m^6^A compared with that of NC-OE. **e** qRT‒PCR analysis of the inhibitory impact of pre-miR-665 and miR-665 on METTL3 overexpression. **f** MeRIP-qPCR showed the negative effect of si-METTL3 on pre-miR-665 methylation by m^6^A compared with that of si-NC. **g** qRT‒PCR analysis of the ability of pre-miR-665 and miR-665 to promote METTL3 inhibition. **h** The mRNA expression of *DSPP, DMP1, ALP, RUNX2* and *OSX* in the SCAPs transfected with miR-665 mimics and inhibitors. **i**, **j** The protein expression of DSPP, DMP1, ALP, RUNX2 and OSX and quantitative analysis in the SCAPs transfected with miR-665 mimics and inhibitors. **k**–**n** Representative images of ALP- and ARS-stained transfected SCAPs at 7 and 14 days in mineralized medium. Scale bar = 200 µm. **o** Immunofluorescence staining results of transfected SCAPs stained for ALP and RUNX2. Scale bar = 40 µm. **p** qRT‒PCR indicated pre-miR-665 was a target of YTHDF2. **q** si-YTHDF2 increased the expression of pre-miR-665 after cotransfection of METTL3-OE. *n* = 3, * indicates *P* < 0.05; ** indicates *P* < 0.01; *** indicates *P* < 0.001.

After identifying the METTL3-mediated promotion of SCAP odonto/osteogenic differentiation, we investigated the molecular mechanism through which METTL3 exerts its regulatory effects. Previous research has suggested that METTL3 can decrease the levels of mature miRNAs by modifying hairpin-shaped precursor miRNAs (pre-miRNAs) with m^6^A, thereby indirectly increasing the expression of miRNA target genes and ultimately participating in the regulation of cell differentiation^23,24^. Hence, to determine the target pre-miRNAs of METTL3, we performed a m^6^A RNA immunoprecipitation (RIP) microarray analysis of METTL3-OE- and NC-OE-infected SCAPs (Fig. 5a). The resulting list of the top 5 m^6^A-methylated pre-miRNAs in SCAPs was compiled. Among these, pre-miR-665 and pre-miR-933 exhibited the highest levels of m^6^A methylation ( > 1.2-fold change) and displayed a high degree of conservation across different species (Fig. 5b, c). Notably, miR-665 was found to promote the transition from acetylation to methylation of the promoters of the odontogenic differentiation markers DSPP and DMP1^25^, suggesting the potential role of this molecule as a repressor during odontoblast induction. Consequently, we screened pre-miR-665 as a promising candidate for further investigation of METTL3 activity.

Subsequently, we investigated the effects of amplifying or suppressing METTL3 on alterations in the m^6^A levels of pre-miR-665 utilizing MeRIP-qPCR. MeRIP-qPCR revealed that increased METTL3 expression led to an increase in the m^6^A methylation level of pre-miR-665, while METTL3 inhibition had the opposite effect (Fig. 5d, f). Furthermore, the expression levels of both pre-miR-665 and miR-665 were notably decreased when METTL3 was overexpressed in SCAPs. In contrast, si-METTL3 increased the production of pre-miR-665 and miR-665, indicating that METTL3-induced pre-miR-665 m^6^A enrichment can suppress miR-665 expression (Fig. 5e, g).

To verify whether METTL3 regulates the odonto/osteogenic differentiation of SCAPs by mediating the m^6^A modification of pre-miR-665, we further examined the impact of miR-665 on the odonto/osteogenic differentiation of SCAPs. Transient transfection of miR-665 mimics and inhibitors was conducted (Supplementary Fig. 4a, b). qRT‒PCR and western blotting demonstrated that miR-665 mimics hindered the odonto/osteoblast differentiation of SCAPs, while miR-665 inhibitors increased the expression of relevant proteins and genes (Fig. 5h‒j). Furthermore, ALP and ARS staining, along with subsequent quantitative analyses, was performed, revealing a diminished degree of odonto/osteogenesis and mineralization in the SCAPs transfected with miR-665 mimics, while miR-665 inhibitors resulted in increased formation of calcium nodules. The immunofluorescence staining results also demonstrated an identical pattern (Fig. 5k–o).

The involvement of YTH domain family proteins, specifically YTHDF2/3 and YTHDC1/2, in m^6^A-related RNA degradation has been demonstrated^26–29^. Consequently, we investigated the presence of collaborative “reader” proteins in the process of METTL3-mediated pre-miR-665 m^6^A methylation in SCAPs. si-YTHDF2 increased the expression of pre-miR-665, indicating that pre-miR-665 may be a potential target of YTHDF2 (Fig. 5p; Supplementary Fig. 4c). Furthermore, the inhibitory effect of METTL3-OE on pre-miR-665 expression was counteracted by cotransfection of SCAPs with si-YTHDF2 (Fig. 5q).

### *DLX3* is directly targeted by miR-665 in SCAPs

**Fig. 6:**
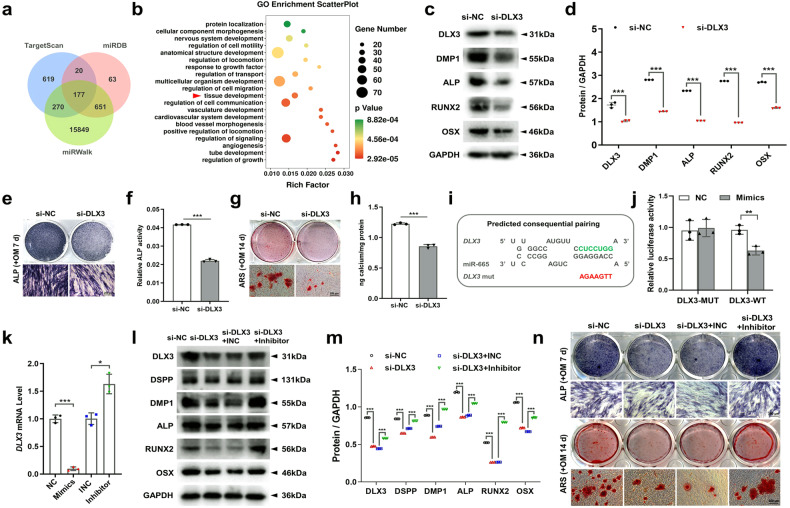
DLX3 acts as a target gene of miR-665. **a** Venn diagram showing the number of miR-665 target genes predicted by the TargetScan, miRDB and miRWalk algorithms. **b** In-depth analysis of 177 target genes through GO analysis. **c**, **d** The protein expression of DMP1, ALP, RUNX2 and OSX in the SCAPs transfected with si-DLX3. **e**–**h** Representative images of ALP staining and ARS staining in the SCAPs transfected with si-DLX3 indicating decreased osteogenic differentiation. Scale bar = 200 µm. **i**, **j** Luciferase reporter assays in 293 T cells indicated that miR-665 could bind to DLX3. **k** qRT‒PCR analysis of the expression of *DLX3* in the SCAPs transfected with miR-665 mimics or inhibitors. **l, m** The inhibition of odonto/osteogenic protein expression caused by si-DLX3 was reversed by cotransfection of SCAPs with miR-665. **n** ARS staining and ALP staining demonstrated that the decrease in calcium nodules induced by si-DLX3 was abrogated by cotransfection with miR-665. Scale bar = 200 µm. *n* = 3, * indicates *P* < 0.05; ** indicates *P* < 0.01; *** indicates *P* < 0.001.

To explore the possible downstream target genes associated with the control of METTL3/miR-665 axis-mediated differentiation, we conducted a computational prediction using the TargetScan, MiRDB, and miRWalk databases to identify candidate target genes. Our analysis revealed a total of 177 potential target genes that bind to miR-665 (Fig. 6a). Moreover, Gene Ontology (GO) analysis indicated a significant correlation between these target genes and diverse cellular biological processes (Fig. 6b). These three databases predicted that *DLX3* can simultaneously bind to miR-665, and GO analysis indicated that *DLX3* is involved in tissue development. Furthermore, previous research has demonstrated the contribution of *DLX3* to the odonto/osteoblastic differentiation of human DPSCs, and *DLX3* mutations have been linked to abnormal tooth development^30,31^. Additionally, a significant association between DLX3 and bone mass has been reported^32^. Interestingly, Heair et al. revealed that miR-665 decreased *DLX3* mRNA expression in dental pulp cells^25^. Therefore, the selection of *DLX3* as a target of miR-665 is justified in SCAPs.

Next, SCAPs were transfected with si-DLX3 (Supplementary Fig. 5a), and our results showed that suppressing DLX3 led to a significant reduction in the odonto/osteoblastic differentiation of SCAPs (Fig. 6c–h). Additionally, a luciferase reporter assay was conducted by introducing wild-type or mutant 3’-UTR sequences into the DLX3 plasmid reporter in 293 T cells. The results revealed that cotransfection of miR-665 with the wild-type DLX3 reporter significantly reduced luciferase activity, while there was no notable alteration when the mutant-type plasmid reporter was utilized (Fig. 6i, j). Intriguingly, the overexpression of miR-665 in SCAPs resulted in a notable reduction in both the DLX3 gene and protein levels compared to those in the cells transfected with the negative control (NC). Conversely, the inhibition of miR-665 had the opposite effect (Fig. 6k; Supplementary Fig. 5b, c). Moreover, the expression of the DLX3 protein decreased upon YTHDF2 knockdown (Supplementary Fig. 5e). This observation was supported by the results obtained from western blotting assays, where the negative impact induced by si-DLX3 was nullified by miR-665 inhibitors (Fig. 6l, m). Furthermore, the restorative effect was convincingly confirmed through ALP staining and ARS staining (Fig. 6n).

### METTL3-mediated regulation of DLX3 mRNA stability depends on m^6^A methyltransferase activity

**Fig. 7:**
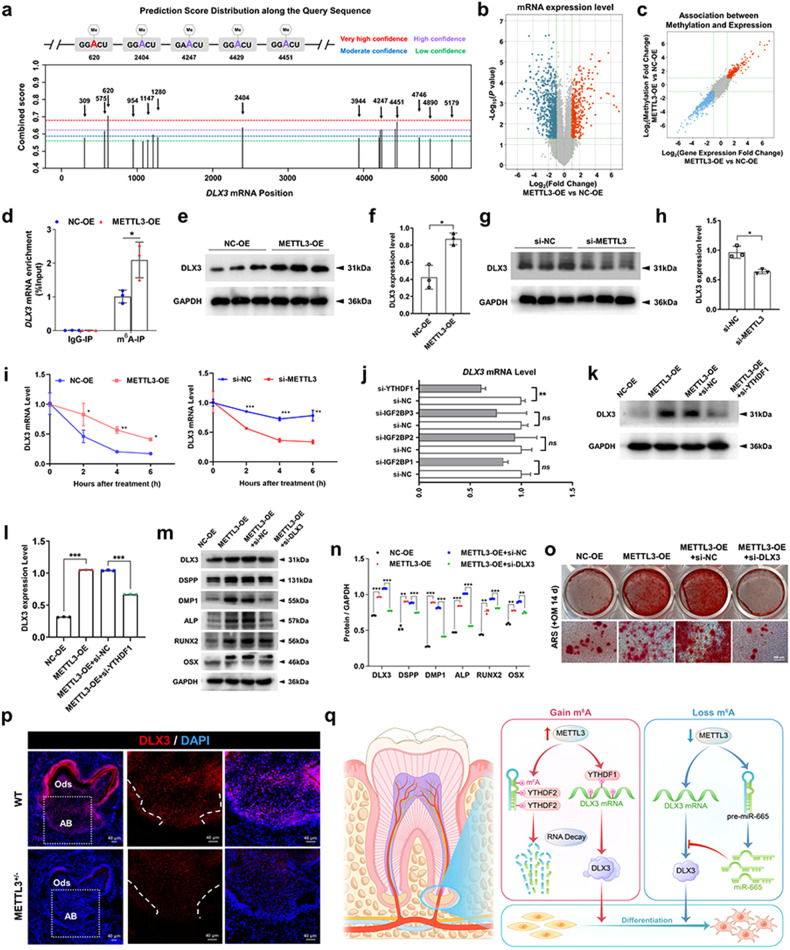
DLX3 serves as a mediator of the odonto/osteogenic action of the METTL3/m^6^A modification axis. **a** Potential sites and regions for m^6^A modification in the sequence of the *DLX3* gene. **b, c** Screening for target genes with increased expression and m^6^A methylation levels in SCAPs after overexpression of METTL3 through m^6^A-RIP microarray sequencing. **d** MeRIP-qPCR illustrated that METTL3 overexpression promoted the m^6^A modification of the *DLX3* gene in SCAPs. **e**–**h** Western blot analysis confirmed a positive correlation between METTL3 and DLX3 expression. **i** The half-life (t_1/2_) of *DLX3* mRNA in the SCAPs in the control, METTL3-OE and si-METTL3 groups was measured by qRT‒PCR. **j** The expression of *DLX3* in the SCAPs transfected with control, si-YTHDF1, si-IGF2BP1, si-IGF2BP2 and si-IGF2BP3 was verified by qRT‒PCR. **k**, **l** Western blotting results showing that the increase in DLX3 expression caused by METTL3-OE was reversed by si-YTHDF1. **m**, **n** si-DLX3 downregulated the expression of odonto/osteogenic protein markers in SCAPs in the presence of METTL3-OE. **o** ARS staining results illustrated that si-DLX3 slightly reversed the generation of mineralized nodules increased by METTL3-OE in SCAPs. Scale bar = 100 µm. **p** Immunofluorescence staining showed that compared with WT mice, METTL3^+/−^ mice exhibited weaker DLX3 expression. Scale bar = 40 µm. **q** A scheme showing the role of the METTL3/pre-miR-665/DLX3 regulatory network in SCAPs (By Figdraw). *n* = 3, ns indicates no significance; * indicates *P* < 0.05; ** indicates *P* < 0.01; *** indicates *P* < 0.001.

Notably, the *DLX3* gene possesses 18 potential m^6^A modification sites, as predicted by a sequence-based m^6^A modification site predictor (Fig. 7a). Additionally, the findings from the m^6^A-RIP microarray analysis demonstrated that the METTL3-OE group exhibited elevated levels of m^6^A methylation and *DLX3* expression compared to the NC-OE group (Fig. 7b, c). To ascertain the direct regulation of *DLX3* m^6^A methylation by METTL3 in SCAPs, we used MeRIP-qPCR. We identified a significant increase in m^6^A enrichment in *DLX3* upon METTL3 overexpression in the m^6^A-RIP group, while no change was observed in the IgG-RIP group (Fig. 7d). Moreover, the excessive expression of METTL3 led to an increase in DLX3 protein levels, while the suppression of METTL3 using si-METTL3 resulted in a reduction in the protein level of DLX3 (Fig. 7e-h). Furthermore, immunofluorescence staining confirmed the decrease in DLX3 expression in the METTL3^+/−^ mice compared to that in their WT littermates (Fig. 7p). To further investigate whether METTL3 regulates DLX3 expression by influencing its mRNA stability, we treated SCAPs with the transcriptional inhibitor actinomycin D. RNA stability curve analysis demonstrated that METTL3-OE increased the stability of *DLX3* mRNA, while si-METTL3 decreased the half-life of *DLX3* mRNA (Fig. 7i).

Given the established role of YTHDF1 and IGF2BPs in promoting the stability of methylated mRNA, qRT‒PCR was used to identify the most effective “reader” protein^18,33^. Based on the observed inhibition of *DLX3* mRNA in the SCAPs transfected with si-YTHDF1 siRNA, we hypothesized that the *DLX3* transcripts are targeted by YTHDF1 (Fig. 7j; Supplementary Fig. 5d). Furthermore, the protein expression of DLX3 in SCAPs was notably reduced by si-YTHDF1 in the presence of METTL3 overexpression (Fig. 7k, l). Moreover, the upregulation of *DLX3* and differentiation-associated genes induced by METTL3 overexpression was effectively counteracted by simultaneous treatment with si-DLX3 (Fig. 7m, n). ARS staining revealed that si-DLX3 decreased the presence of calcium nodules in the SCAPs of the METTL3-OE group (Fig. 7o). Based on these findings, we can infer that *DLX3* is a functionally regulated target of METTL3, and its effects are dependent on YTHDF1.

As depicted in the Graphical Abstract, the odonto/osteogenic differentiation capabilities of SCAPs were significantly augmented as a result of the upregulation of METTL3. Mechanistically, METTL3 facilitated the m^6^A modification of pre-miR-665, which was subsequently recognized by YTHDF2, leading to its degradation and consequent suppression of miR-665 expression. As a result of this inhibition, the target gene *DLX3* was ultimately expressed more strongly, thus facilitating the differentiation of SCAPs into odonto/osteogenic cells. Additionally, METTL3 directly mediates the m^6^A modification of *DLX3*. Methylated *DLX3* was subsequently recognized by another m^6^A “reader”, YTHDF1, to ensure its stability and expression (Fig. 7q).

## Discussion

Stem cells are integral to maintaining tissue homeostasis and facilitating regeneration. These cells remain in a dormant state within a designated microenvironment for a long period of time, during which epigenetic alterations contribute to modifications in their niche environment and the regulation of their differentiation trajectory^34^. SCAPs obtained from the outer region of the root foramen exhibit strong proliferative activity and the ability to differentiate into multiple lineages, all of which are closely linked to root development^9,14^. In addition, SCAPs exhibit increased osteoblastic potential^35^. Therefore, SCAPs are crucial progenitor cells for the regeneration of tooth roots and bone tissues.

The reversibility and dynamic nature of ubiquitous m^6^A methylation have been demonstrated, and this process is influenced by methyltransferases, demethylases, and methylated readers. Methyltransferases, including METTL3, METTL14, and WTAP, primarily facilitate the progression of m^6^A binding. The METTL3/METTL14 complex serves as the central component of a larger complex responsible for the internal installation of m^6^A^36^. Recently, other rare methyltransferases, such as METTL5, METTL16, ZC3H13, and ZCCHC4, have been identified^37^. Demethylases primarily fall under the AlkB-related family, which includes FTO and ALKBH5. These demethylases serve as counterparts to methyltransferases and are responsible for eliminating m^6^A modifications^38^. Consequently, the dynamic increase in relevant m^6^A modifications is regulated by methylation readers, thereby activating subsequent biological processes such as RNA stability control, transcription and translation, and selective splicing. m^6^A readers, including YT521-B homology (YTH) domain family proteins (YTHDF1/2/3, YTHDC1/2) and insulin-like growth factor-2 binding protein family proteins (IGF2BP1/2/3), are usually referred to as executors^39,40^.

Studies have presented compelling evidence supporting a strong and intricate association between METTL3-mediated m^6^A modification and the regulation of stem cells. For example, Wu et al. and Yan et al. successfully demonstrated the critical impact of METTL3-mediated m^6^A modification on osteogenic outcomes^23,41^. Our findings showed that METTL3 can augment both odontogenic and osteogenic differentiation of SCAPs, indicating the potential utility of METTL3 as a novel strategy for regenerating biological tooth roots and maxillofacial bone tissues using SCAPs. However, notably, some reports have identified a negative effect of METTL3 modifications on osteoblast differentiation in stem cells^42^. This dual role of METTL3 and m^6^A modification in osteogenic differentiation may be attributed, at least in part, to variations in the cell lines used in different studies, suggesting the complexity of m^6^A modification.

Recent research has shown that methyltransferases can independently regulate target genes, independent of their role in m^6^A modification. Su et al. specifically showed that METTL16 promotes translation in the cytosol via its MTase domain rather than through m^6^A modifications^43^. Notably, while the overall level of m^6^A remained consistent among the various groups, the persistence of m^6^A modifications was notable. For example, Ye et al. reported a close association between YTHDF1 and hypopharyngeal squamous cell carcinoma, where the global RNA m^6^A level did not significantly change between the YTHDF1 knockdown and control groups. However, YTHDF1 was found to regulate TFRC synthesis by interacting with its m^6^A mRNA^44^. In this study, the results from the quantification of m^6^A and m^6^A dot blot analysis demonstrated an increase in m^6^A levels at Days 3 and 7 during the mineralization induction of SCAPs. Furthermore, analysis of m^6^A modifications via MeRIP-qPCR and m^6^A dot blot revealed significant differences in the enrichment zone upon manipulation of METTL3 expression through overexpression or silencing. Overall, these results provide evidence for the regulatory role of METTL3 in the m^6^A-dependent directional differentiation of SCAPs.

MicroRNAs (miRNAs) play a crucial role in regulating a wide range of biological processes, including cellular pathways, embryonic development, and tissue homeostasis^45,46^. The biogenesis of miRNAs begins with the transcription of primary RNAs (pri-RNAs), which are subsequently processed in the nucleus by the microprocessor complex comprising the RNase III enzyme Drosha and the double-stranded RNA-binding protein DGCR8. This processing results in the formation of hairpin-shaped RNAs known as precursor RNAs (pre-miRNAs). Pre-miRNAs are subsequently translocated to the cytoplasm, where the RNase III DICER1 associates with pre-miRNAs and produces a double-stranded miRNA intermediate derived from the stem of the pre-miRNA hairpins. Subsequently, one strand is preferentially chosen and incorporated into the RNA-induced silencing complex (RISC) with the assistance of a cascade of effectors^47,48^. Ultimately, mature miRNAs direct the RISC toward complementary regions on target mRNAs, leading to the degradation of these target mRNAs. The selective control of the directional differentiation of SCAPs by miR-143-3p or miR-497-5p through distinct target genes has been confirmed^49,50^. However, the effect of epigenetic modifications during miRNA maturation on the biological characteristics of SCAPs has been overlooked in these studies. Recent studies have highlighted the role of m^6^A modification in the regulation of mature miRNA formation and its subsequent impact on gene expression. Yan et al. discovered that METTL3-mediated m^6^A modification specifically targets pre-miR-320, thereby contributing to the regulation of directional differentiation in BMSCs^23^. In this study, pre-miR-665 was screened as a potential target using a m^6^A RNA RIP microarray, followed by MeRIP-qPCR and gain- and loss-of-function analysis for further validation. Our study demonstrated the involvement of METTL3/pre-miR-665 signaling pathways in the committed differentiation of SCAPs.

*DLX3*, a member of the *DLX* family of transcription factors, plays an important role in the deposition of the mineral matrix and biomineralization, particularly in the development of bone and teeth^31^. Mutations in *DLX3* have been identified as the cause of a rare ectodermal dysplasia known as tricho-dento-osseous (TDO) syndrome, which is characterized by abnormal hair, tooth, and bone features, suggesting disrupted interactions between epithelial and mesenchymal tissues^51^. Several studies have demonstrated the impact of DLX3 on odontogenesis and osteogenesis through its targeted regulation of DSPP and RUNX2 expression^30,32^. However, the methylation of *DLX3* mRNA via m^6^A modification has not been previously reported. Although Heair et al. explored the role of DLX3 in dental pulp stem cells^25^, our findings revealed its novel function in SCAPs. This study provides novel insights into the role of *DLX3* in METTL3-mediated modification and pre-miR-665 in the regulation of the committed differentiation of SCAPs.

In conclusion, the present study demonstrated that METTL3-mediated m^6^A modification of pre-miR-665/DLX3 is essential for regulating the odontogenic/osteoblastic differentiation of SCAPs. These findings present a promising and viable alternative target for clinical applications in biological root and bone regeneration.

### Supplementary information

Supplementary Information

## Data Availability

The data of this study are available from the corresponding authors upon reasonable request.
