# Supplementary material for: METTL3-mediated pre-miR-665/DLX3 m6A methylation facilitates the committed differentiation of stem cells from apical papilla
Source: Exp Mol Med. 2024 Jun 3;56(6):1426–38. doi: 10.1038/s12276-024-01245-8 (PMC11263550; doi:10.1038/s12276-024-01245-8)
Supplement: Supplementary file 1 — Supplementary Information [file 12276_2024_1245_MOESM1_ESM.pdf]

Supplementary Figures

Supplementary Fig. 1

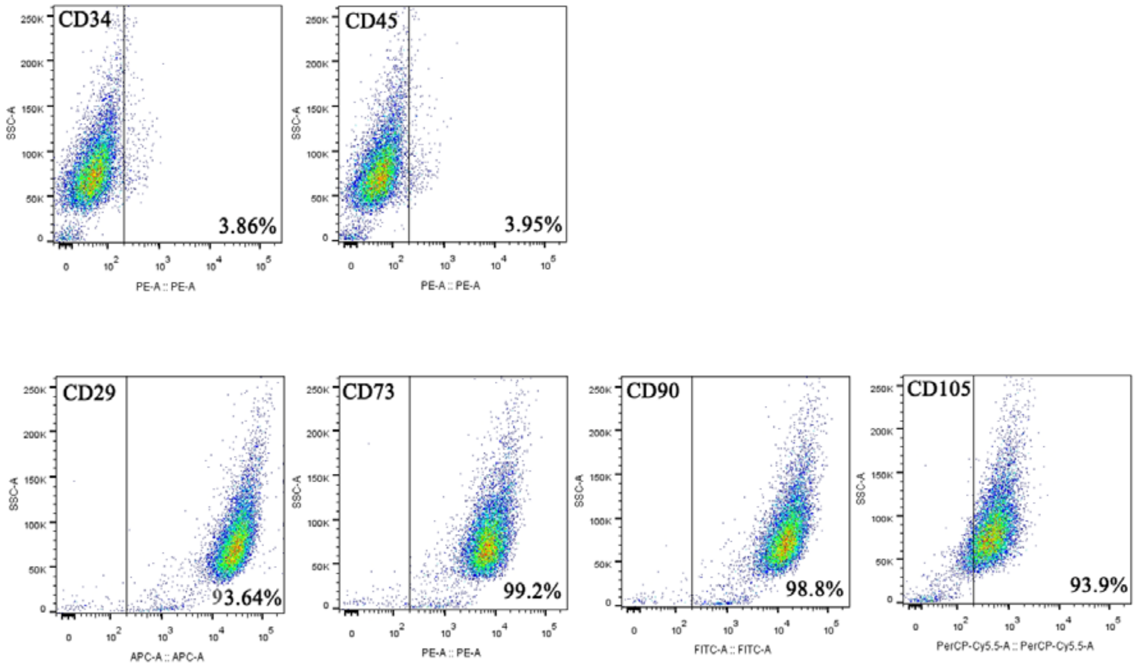

Supplementary Fig. 2

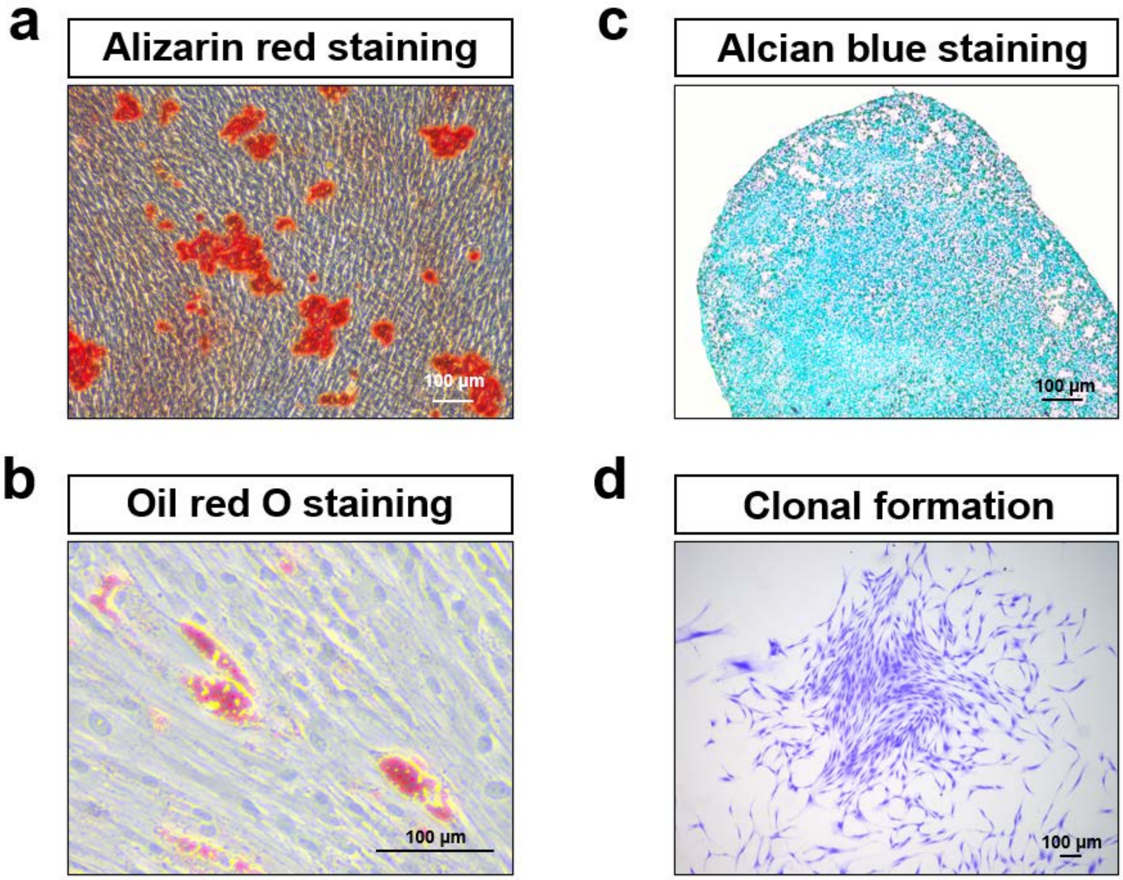

Supplementary Fig. 3

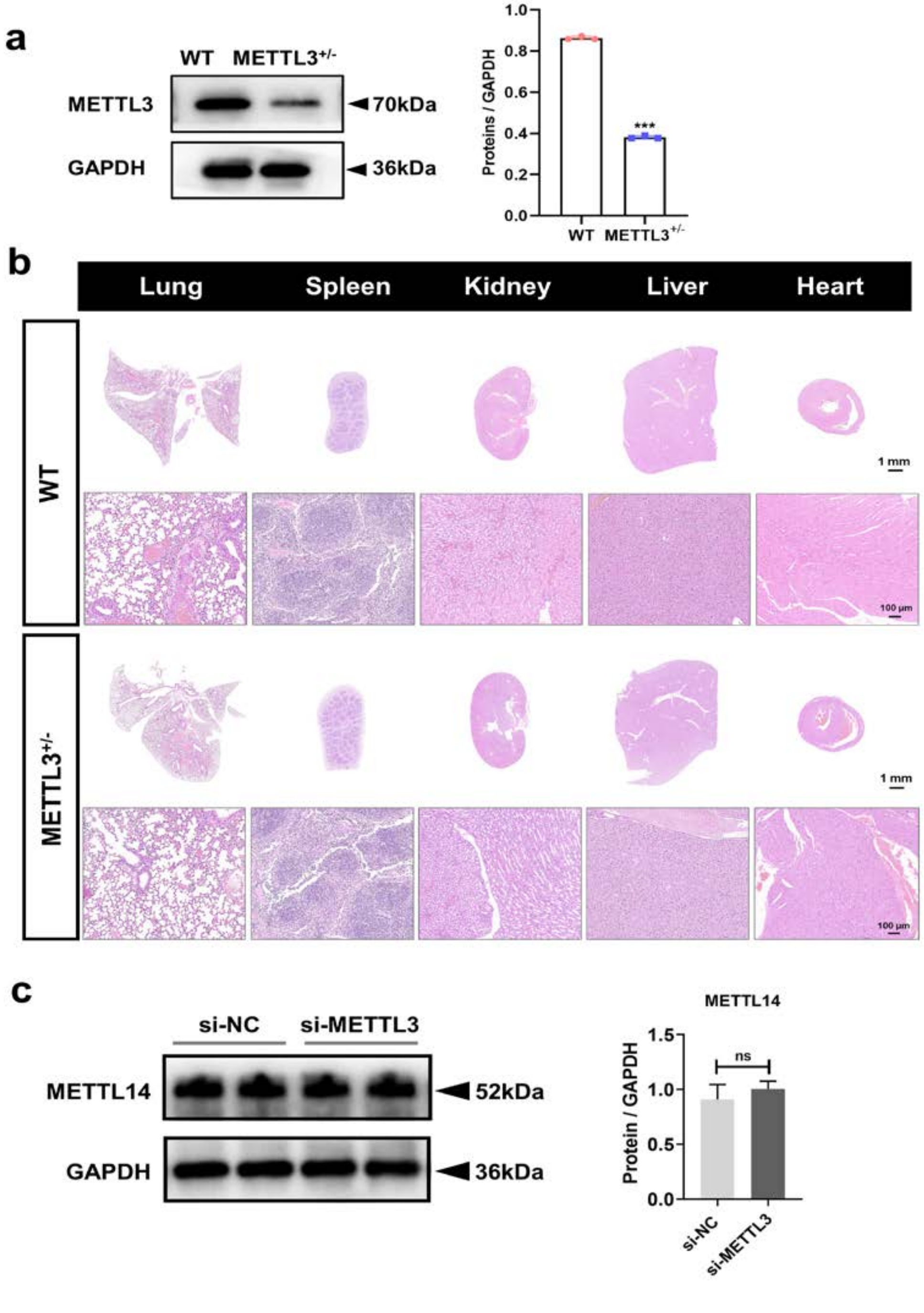

Supplementary Fig. 4

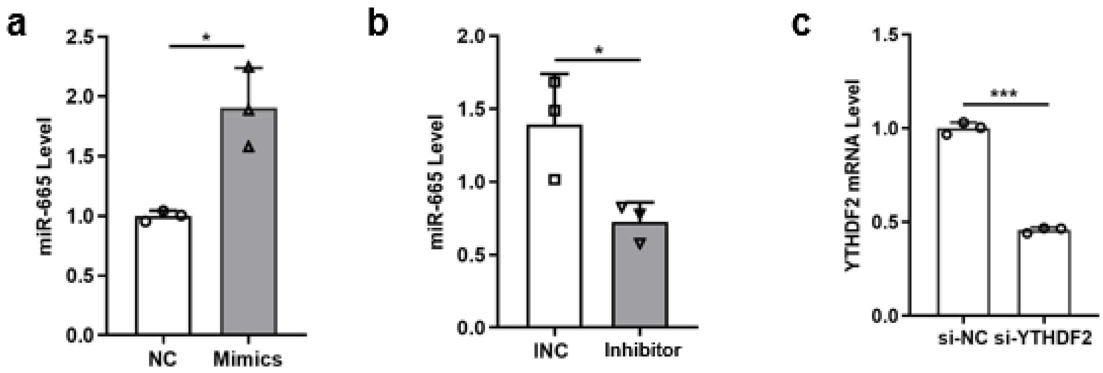

Supplementary Fig. 5

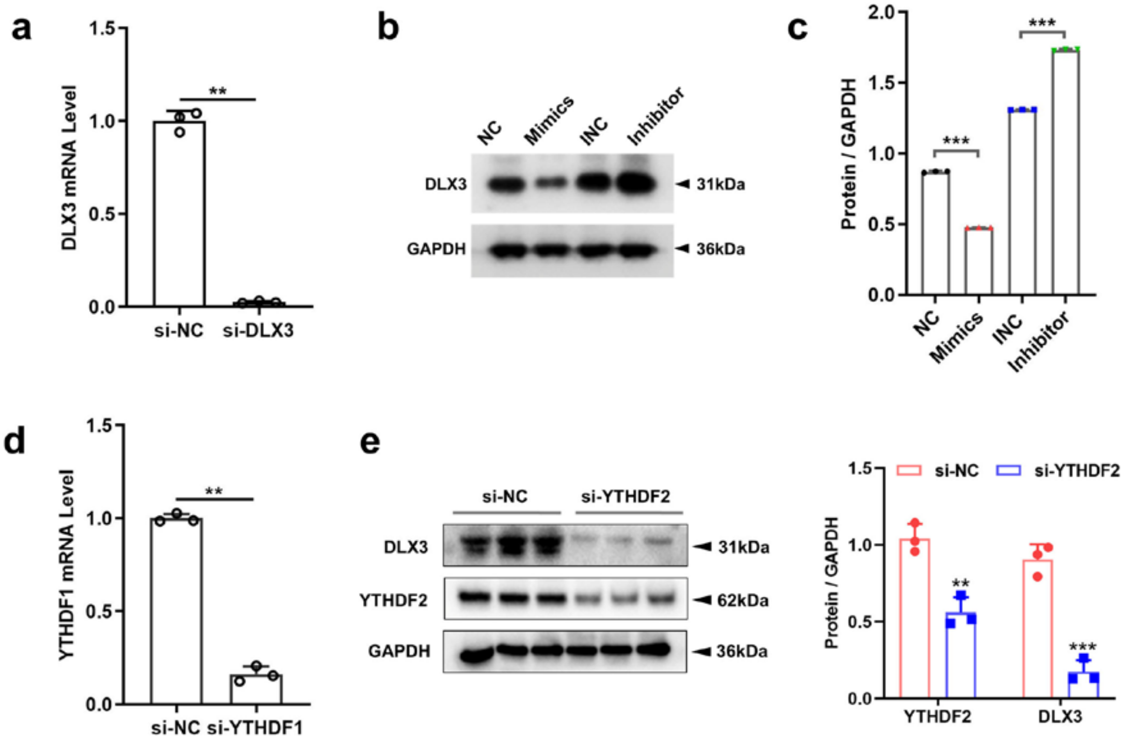

Supplementary Fig. 6

Fig. 1

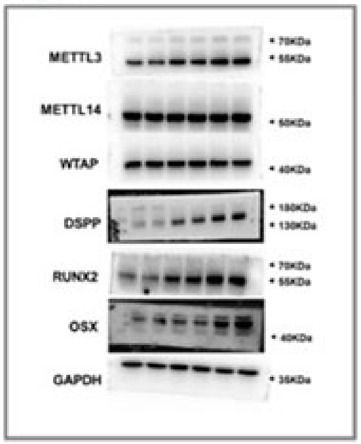

Fig. 3

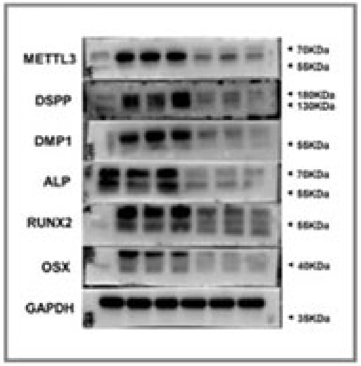

Fig. 4

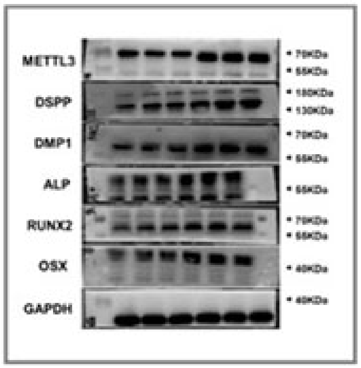

Fig. 5

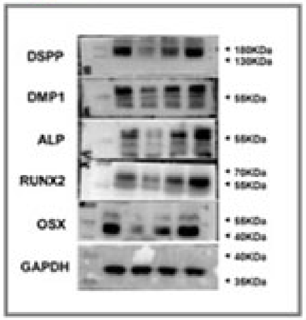

Fig. 6

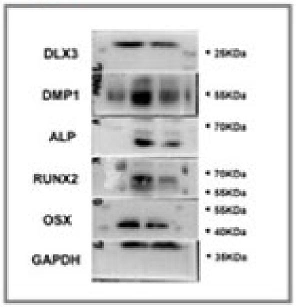

Fig. 6

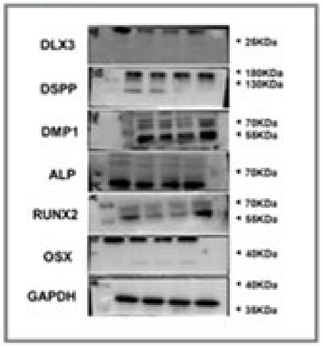

Fig. 7

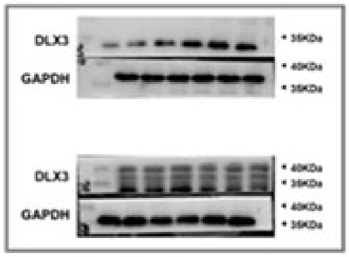

Fig. 7

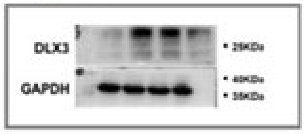

Fig. 7

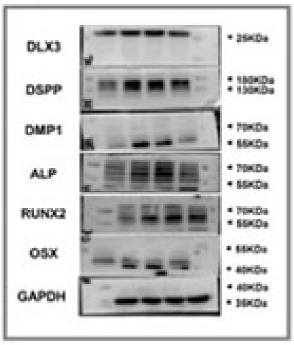

Supplementary Tables

Supplementary Table 1. The primer sequences used for real-time PCR

Gene	Forward primers (5'-3')	Reverse primers (5'-3')
METTL3	ACCCTGACAGATGATGAGATGC	CGTTCATACCCCCAGAGGTTTAG
DSPP	ATATTGAGGGCTGGAATGGGGA	TTTGTGGCTCCAGCATTGTCA
DMP1	AGGAAGTCTCGCATCTCAGAG	TGGAGTTGCTGTTTTCTGTAGAG
RUNX2	TCTTAGAACAAATTCTGCCCTTT	TGCTTTGGTCTTGAAATCACA
OSX	CCTCCTCAGCTCACCTTCTC	GTTGGGAGCCCAAATAGAAA
ALP	GGACGCTGGGAAATCTGTG	CCATGATCACGTCAATGTCC
YTHDF1	ACCTGTCCAGCTATTACCCG	TGGTGAGGTATGGAATCGGAG
YTHDF2	TGTTGGAGAAGCTTCGGTCC	ACCCGGCCATGTTTCAGATT
YTHDF3	ATCAGAGTAACAGCTATCCAC	CCCAGGTTGACTAAATACAC
YTHDC1	AACTGGTTTCTAAGCCACTGAGC	GGAGGCACTACTTGATAGACGA
YTHDC2	CAAAACATGCTGTTAGGAGCCT	CCACTTGTCTTGCTCATTTCCC
IGF2BP1	GCGGCCAGTTCTTGGTCAA	TTGGGCACCGAATGTTCAATC
IGF2BP2	AGCTAAGCGGGCATCAGTTTG	CCGCAGCGGGAAATCAATCT
IGF2BP3	TATATCGGAAACCTCAGCGAGA	GGACCGAGTGCTCAACTTCT
GAPDH	GGAGCGAGATCCCTCCAAAAT	GGCTGTTGTCATACTTCTCATGG
U6	CTCGCTTCGGCAGCACA	AACGCTTCACGAATTTGCGT

Supplemental Table 2. The oligonucleotide sequences used in the study

The oligonucleotide name	Sense sequences (5'-3')
si-METTL3	CAAGTATGTTCACTATGAA
si-DLX3	CCACCAACTCCTGGTATCA
si-YTHDF1	CTCCACCCATAAAGCATAA
si-YTHDF2	GACCAAGAATGGCATTGCA
miR-665 mimic-NC	UUUGUACUACACAAAAGUACUG
miR-665 mimic	ACCAGGAGGCUGAGGCCCCU
miR-665 inhibitor-NC	CAGUACUUUUGUGUAGUACAAA
miR-665 inhibitor	AGGGGCCUCAGCCUCCUGGU

Supplemental Figure Legends

Supplementary Fig.1. Flow cytometry analysis indicated that SCAP were positive for CD29, CD73, CD90 and CD105 and negative for CD34 and CD45; n=3

Supplementary Fig. 2. Representative images of alizarin red staining, oil red O staining and alcian blue staining showed the capacity of SCAP to multi-differentiate; Clonal Formation assay showed the capacity to proliferate of SCAP. Scale bar = 100 µm.

Supplementary Fig. 3. a The genotype was verified by western blotting assay. **b** Representative images of HE staining indicated that size and function of lung, spleen, kidney, liver and heart were no different in METTL3 $^{+/-}$ mice compared with controls. **c** Western blotting detection of the effect of knocking down METTL3 on the expression level of METTL14; n=3. Scale bar = 1mm and 100µm.

Supplementary Fig. 4. The results of qRT-PCR. **a** The efficiency of miR-665 overexpression. **b** Knockdown of miR-665.**c** Knockdown of YTHDF2; n=3. * indicates $P < 0.05$; *** indicates $P < 0.001$.

Supplementary Fig. 5. DLX3 acts a target gene of miR-665 and stabilized by YTHDF1. **a** si-DLX3 had a good efficiency according to qRT-PCR; n=3. **b, c** miR-665 mimics reduced DLX3 protein level while miR-665 inhibitors promoted DLX3 protein level. GAPDH was used as an internal control; n = 3. **d** The efficiency of si-YTHDF1 was examined by qRT-PCR. **e** Western blot detection of the effect of knocking down YTHDF2 on the expression level of DLX3; n=3. ** indicates $P < 0.01$; *** indicates $P < 0.001$.

Supplementary Fig. 6. The original data of western blotting assay.
